# Outcomes of a hydroxyapatite ceramic-coated femoral stem in primary total hip arthroplasty: a report of excellent survivorship from a single United Kingdom centre

**DOI:** 10.1051/sicotj/2024026

**Published:** 2024-08-14

**Authors:** Karim M. Abdelghafour, Sherif A. Khaled, Khaled F. M. Abdel-Kader, Hazem A. Azeem, Nirav N. Shah

**Affiliations:** 1 Worthing Hospital, University Hospitals of Sussex Lyndhurst Road Worthing BN112DH UK; 2 Kasr Alainy School of Medicine Cairo University Al-Saray Street El Manial Cairo 11956 Egypt

**Keywords:** Hydroxyapatite-coated stems, Oxford Hip Score, Total hip arthroplasty, Survival, Osseointegration

## Abstract

*Background*: Hydroxyapatite (HA) coated femoral stems were introduced to enhance the biological fixation at the implant-bone interface, aiming to increase the longevity and survival of the prostheses. We aimed to assess the long-term outcomes of an HA ceramic (HAC) coated stem in primary total hip arthroplasty (THA), assess the stem survival, and clinically evaluate the patients using patient-reported outcome measures (PROMs) and radiological evaluation of stem osseointegration. *Patients and methods*: This was a prospective evaluation of a retrospective cohort of 385 patients (442 hips) who underwent primary THA between June 2008 and December 2018. The mean age was 63.83 years (range, 30–82 years). During the follow-up duration, 23 patients died, and 36 patients (38 hips) were lost to follow-up. Prospective data collected for 326 patients (381 hips) was used to evaluate stem survival with the Kaplan–Meier method using aseptic loosening or any revision as the endpoint. Clinical evaluation was done using the EuroQol five-dimension (EQ-5D) scoring system and PROMs using the Oxford Hip Score (OHS) and Merle D’Aubigne Postel (MDP) score. Radiological assessments were performed using the Engh radiological criteria for stem osteointegration. *Results*: The mean follow-up duration was 9.39 years (range, 4–14.5 years). The survival of the HAC-coated femoral stem was 100% (95% confidence interval [CI], 96.7–100%) at 14 years with aseptic loosening as the endpoint, and 98.9% (CI, 96.7–100%) at 14 years with stem revision for any reason as the endpoint. The mean OHS was 44.5 (range, 30–48), and the mean MDP score was 15.87 (range, 10–18). Radiological evaluations showed full osseointegration of all stems. *Conclusion*: This HAC-coated femoral stem has shown excellent survivorship, functional outcomes, and full osseointegration at the final follow-up.

## Introduction

The dramatic pain relief and restoration of function achieved by total hip arthroplasty (THA) make the procedure life-changing for patients with degenerative hip disease, owing to the impact on the patient’s quality of life. However, THA can carry a risk of complications such as loosening, wear, periprosthetic fractures, and dislocation [1, 2]. With the increasing life expectancy among the population and younger patients requiring THA, there is an increased use of uncemented prostheses for THA. The longevity of the femoral stem used in THA has become crucial to ensure excellent outcomes and patient satisfaction [3, 4].

Hydroxyapatite (HA) coatings were introduced to enhance the biological fixation of hip prostheses owing to the osteoconductive and osteoinductive properties [5]. HA promotes bone growth at the implant-bone interface without intervening fibrous tissue formation. Another key characteristic of HA is crystallinity, which is associated with increased bioactivity, bone growth, and decreased bone resorption [6].

**Figure 1:**
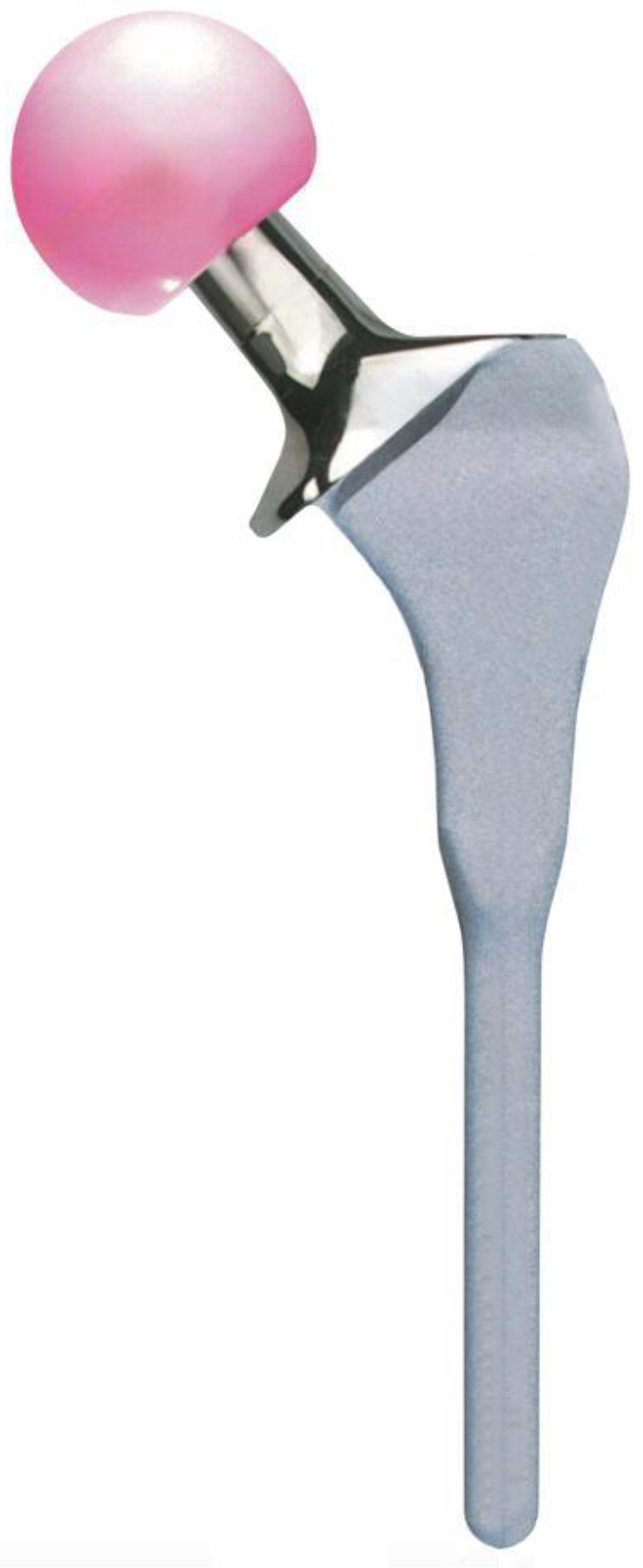
Furlong HAC stem design.

The Furlong hydroxyapatite ceramic (HAC) femoral stem (JRI Orthopaedics Ltd, London, UK) was the first-ever HAC stem used in THA in 1985. It is manufactured from a titanium alloy (Ti-6Al-4V), with its surface plasma-sprayed with a 200-μm thick layer of HA with high crystallinity. The stem is collared and designed to achieve primary stability by proximal metaphyseal fit (Figure 1), and the rectangular cross-section of the proximal body provides rotational stability under dynamic loading. The cone-shaped geometric transition between the proximal body and cylindrical distal stem is intended to discourage subsidence [7].

We aimed to assess the long-term results of this HAC-coated femoral stem in primary THA. We assessed the survival of the stem at 14 years by using aseptic loosening or any revision as the endpoint, with clinical evaluation and radiological evaluation of the osseointegration of the stem.

## Patients and methods

Between June 2008 and December 2018, a cohort of 385 patients (442 hips) had THA performed using the Furlong HAC-coated femoral stem. Evaluation of data for this retrospective series was done. We included all patients who had primary THA during this period with at least 4 years of follow-up. Patients who lacked the mental capacity, revision arthroplasty cases, and patients with <4 years of follow-up were excluded.

### Surgical technique

The surgical procedure was standardised, and all procedures were performed by or under the direct supervision of a single surgeon via a posterior approach to the hip with the patient in the lateral position. The CSF Plus HAC-coated cup (JRI Ltd, London, UK) was used in all cases. A modular 36 mm fourth-generation ceramic head (Biolox Delta) was used in all hips on a ceramic liner.

Prophylactic antibiotics were administered according to the local guidelines. Venous thromboprophylaxis in the form of low molecular weight heparin was given postoperatively. Patients were allowed full weight bearing postoperatively, and all patients had physiotherapy before discharge.

### Clinical and radiological evaluation

Follow-ups were performed at 6 weeks, 12 weeks, 6 months and 12 months postoperatively, and 5 years. During the Coronavirus disease 2019 pandemic, some follow-ups were done using telephone consultations to avoid patient exposure to infection risk. Patients who were unable to attend follow-ups completed postal questionnaires.

Patient-reported outcome measures (PROMs) were collected, and clinical assessments of pain, mobility, and function were performed using the Merle d’Aubigné and Postel (MDP) scoring system and Oxford Hip Score (OHS) assessing pain and functional ability from the patient’s perspective [8]. We assessed health-related quality of life using the EuroQol-5D visual analogue score, which ranges from 0 to 100 [9]. We assessed the presence of anterior thigh pain, any dislocation events, or periprosthetic fractures. The incidence of infection, thromboembolic complications, and any bearing-related complications were also recorded.

Radiological evaluation was carried out by two independent reviewers to provide inter-observer reliability. Gruen zones for the femur and DeLee and Charnley zones for the acetabulum were used for the assessment of any migration, subsidence, and stress shielding. Stability and osseointegration were assessed using the Engh radiological criteria for uncemented stems [10]. Heterotopic ossification (HO) was identified and graded according to Brooker classification [11].

Data analyses were performed using the Statistical Package for Social Sciences (SPSS) version 28.0 (SPSS Inc., Chicago, Illinois). Cumulative survival analysis using the Kaplan–Meier method was done with corresponding 95% confidence intervals. The endpoint was the revision of the femoral component for any reason or aseptic loosening, and the date of the latest follow-up was defined as a censored event. A *P*-value ≤0.05 was considered statistically significant.

## Results

From 385 patients (442 hips) who met the inclusion criteria, 23 patients (23 hips) died from causes unrelated to the THA. By tracking those patients’ general practitioner records, none of the patients reported any clinical problems with their hips within 1 year of their death. In total, 33 patients (38 hips) were excluded because of cognitive impairment, dementia, or being lost to follow-up. The number of patients who participated in the study was 326 patients, of which 55 had bilateral THAs, and the number of hips available for final follow-up was 381 hips.

The mean follow-up duration was 9.39 years (range, 4–14.5 years). The mean age at the time of the procedure was 63.83 years (range, 30–82 years). Surgical indications and patient demographics are detailed in Table 1.

**Table 1:** Patient demographics.

Characteristic	*n*
Patients, *n*	326
THA, *n*	381
Sex	
Women, *n* (%)	174 (53.4)
Men, *n* (%)	152
Mean age, years (range)	63.83 (30–82)
Mean follow-up duration, years (range)	9.39 (4–14.5)
BMI, kg/m^2^ (range)	27.73 (16.5–49)
Indication for THA, n (%)	
OA	314 (82.6)
AVN	24 (6.2)
Dysplasia	26 (6.8)
Rheumatoid arthritis	12 (3.1)
Trauma	4 (1)
Ankylosing spondylitis	1 (0.3)

THA, total hip arthroplasty; BMI, body mass index; OA, osteoarthritis; AVN, avascular necrosis.

### Clinical evaluation and stem survival

The mean visual analogue score (VAS) of the EuroQol-5D was 83.46 (range, 40–100), with 348 (91.3%) hips having a score ≥70. The mean MDP score for the 381 hips was 15.87 (range, 10–18). The mean OHS was 44.5 (range, 30–48), with improvement compared to the mean pre-operative OHS, which was 19.31 (*P* = 0.02). We found a correlation between the OHS and comorbidities affecting mobility, such as spinal pathology, stroke, or adjacent joint arthritis. There were 25 patients with an OHS < 39; 16 of them (64%) had these comorbidities. For the patients with an OHS ≥39, 89% did not have these comorbidities with a statistically significant difference (*P* = 0.018).

**Figure 2:**
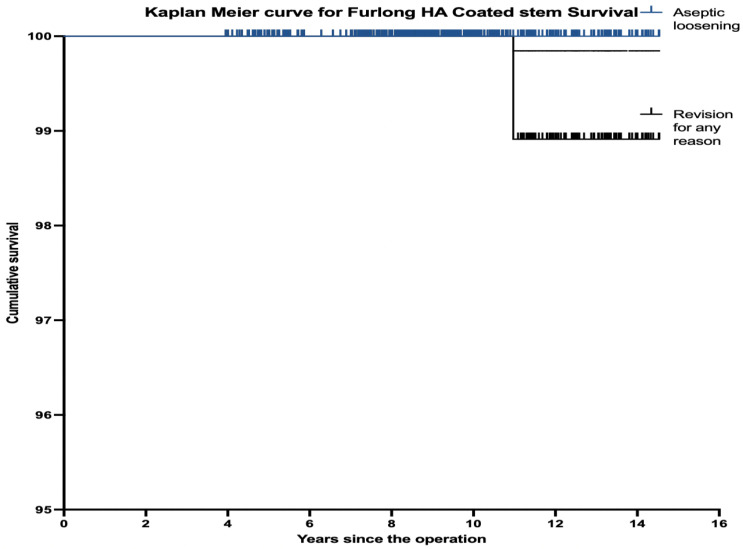
Kaplan–Meier survival curve for HA-coated stem survival.

**Figure 3:**
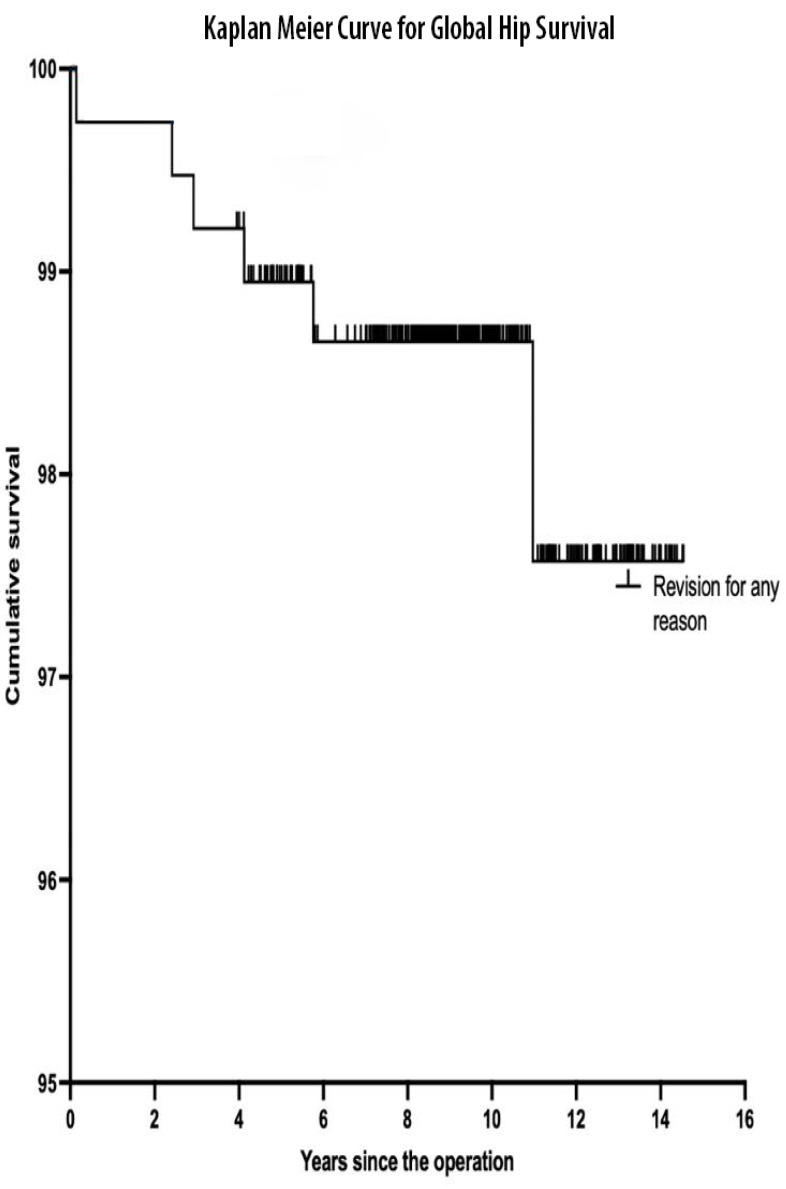
Kaplan–Meier curve for whole THA survival.

The cumulative survival for the HAC-coated femoral stem was 100% (95% CI, 96.7–100%) with aseptic loosening used as the endpoint, and 98.9% (95% CI, 96.7–100%) with revision for any reason as the endpoint (Figure 2). The survival for the whole THA was 97.6% (95% CI, 95.2–100%) with a mean follow-up duration of 9.39 years using any revision as an endpoint (Figure 3).

### Radiological evaluation

**Figure 4:**
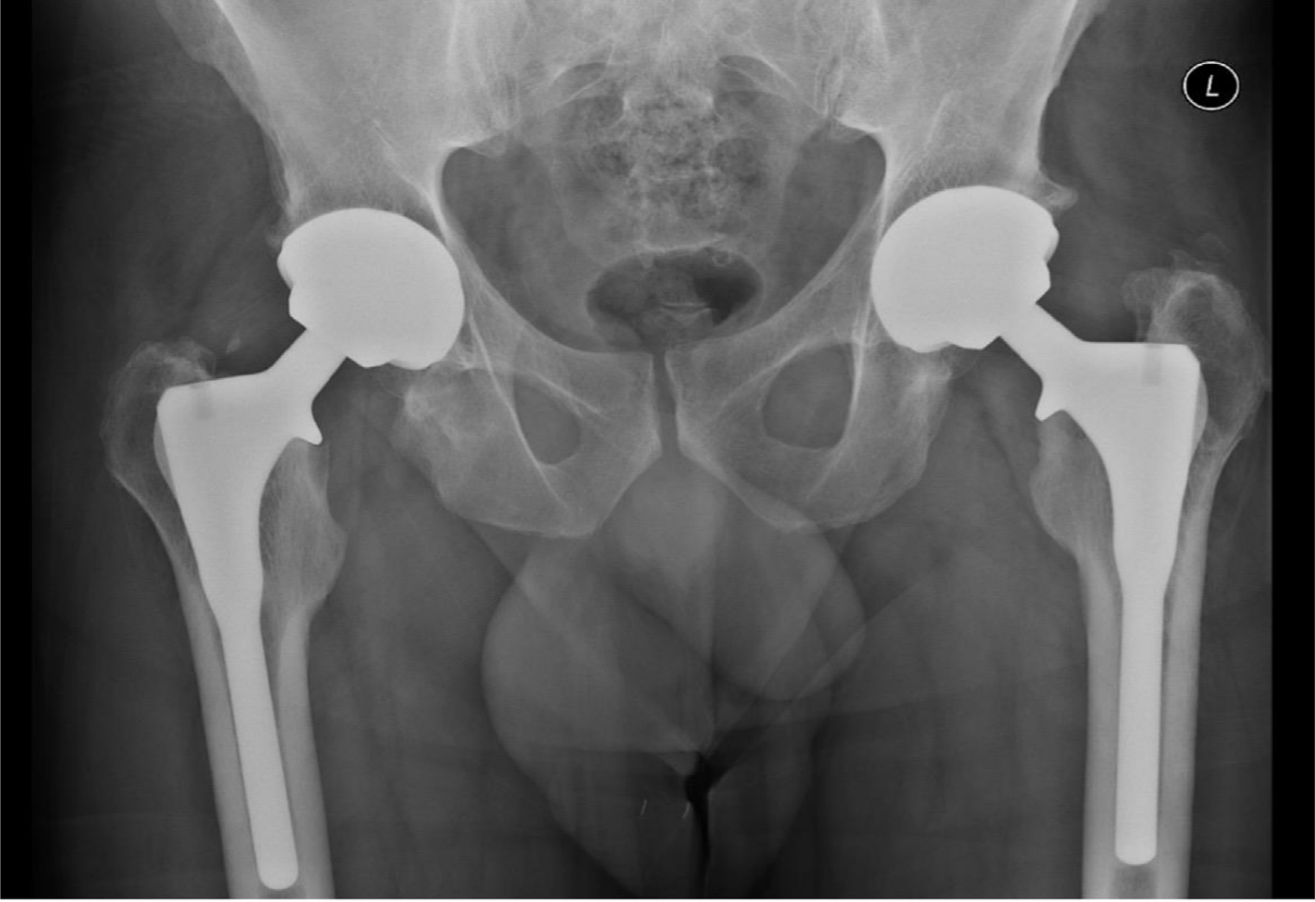
Pelvis anterior-posterior radiographs 12 years postoperatively with evidence of full osteointegration of the stems with spot welds.

**Figure 5:**
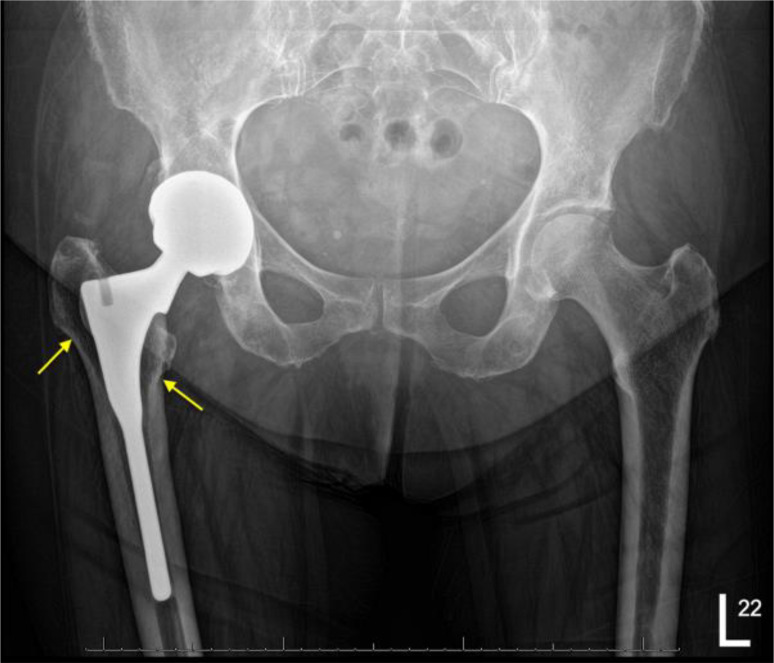
A 10-year post-operative radiograph with lucency at Gruen zones 1 and 7 (arrows) with no clinical signs or pain reported.

Radiological evaluation was done for 301 patients (356 hips) who were able to attend for radiographs at the final follow-up*,* with a mean Engh score of 18.425 (range, 7–27), revealing osseointegration of all stems (Figure 4). Two hips (0.5%) showed subsidence of 3 mm within 1 year postoperatively, but subsequent serial radiographs showed no further subsidence, with radiological evidence of complete osseointegration. There were 17 stems (4.4%) that showed some bone remodelling in the form of stress shielding, particularly at Gruen zones 1, 2, and 7. None of these patients complained of any pain, and by following their radiographs, there were no signs of stem migration (Figure 5).

### Complications and secondary surgeries

In total, seven patients required revision arthroplasty, one patient underwent stem revision due to deep prosthetic joint infection (PJI), and six patients required acetabular revisions. Among the acetabular revisions, one was performed for PJI, two due to failure of osseointegration, two cups were revised due to malpositioning, and one cup was revised following a periprosthetic acetabular fracture. Other complications included two cases that required abductor repair for abductor dysfunction, two cases that developed early post-operative infection treated by washout and implant retention, and one patient that required open reduction and internal fixation for periprosthetic fracture Vancouver type B1. There were eight intra-operative fractures (2%) (seven calcar splits and one greater trochanter fracture) that required cabling or cerclage wires, and none of the cases required the use of revision implants instead of the planned HAC-coated stem. Two cases experienced Vancouver AG periprosthetic fractures that were treated conservatively.

There were two dislocations (0.5%) in our study; one patient had dislocation 4 weeks postoperatively, and the hip was relocated with no instability issues afterwards, the second case required revision of the acetabular component. Seven cases (1.8%) had deep venous thrombosis (DVT), and two patients (0.5%) developed pulmonary embolism (PE); all were treated with anticoagulation and none of the cases had mortality. Despite the radiological findings of HO in 139 hips, the patients who complained of stiffness from the HO were 10 (2.6%), with two patients only requiring surgical excision of the HO. Four cases (1%) complained of iliopsoas impingement, 13 patients (3.4%) were diagnosed with trochanteric bursitis for lateral hip pain, and two (0.5%) patients complained about thigh pain (Table 2).

**Table 2:** Incidence of complications in the study.

Complication	Number	%
Revision arthroplasty	7	1.8
Infection	3	0.8
Fractures: Femoral/acetabular(intra-operative/periprosthetic)	12	3.1
Dislocation	2	0.5
Subsidence	2	0.5
Heterotopic ossification	10	2.6
DVT/PE	9	2.3
Iliopsoas impingement	4	1
Lateral hip pain (trochanteric bursitis)	13	3.4
Thigh pain	2	0.5

DVT/PE, deep vein thrombosis/pulmonary embolism.

## Discussion

Our study showed excellent survival rates for this HAC-coated femoral stem with a mean follow-up duration of 9.39 years, with 100% survival with aseptic loosening as the endpoint and 98.9% with stem revision due to any reason. These findings are comparable with other studies assessing this HAC-coated stem [4, 12, 13]. To our knowledge, this is the largest study assessing the outcomes of this stem, wherein we unified the bearing system to limit the bias from using different bearing systems on the survival rates, and ceramic on ceramic bearings were used owing to the superior wear properties and lower wear and osteolysis rates than traditional bearing surfaces [14].

The survival results were comparable to other studies assessing different HA-coated stems [15–17]. The stem used in our study now has the highest Orthopaedics Device Evaluation Panel (ODEP) rating, which is 15A*. The ODEP was set up to evaluate the outcomes of arthroplasty prostheses and ensure compliance with the benchmarks set by the National Institute of Clinical Excellence (NICE) [18].

The clinical evaluation revealed significant improvement in the OHS and MDP scoring systems and improvement in the quality of life following the procedure. The results are comparable with other studies that used the same scoring systems [4, 12, 16] and other scoring systems for hip function [13, 15, 17]. The decline of OHS reported over the years by some patients may be explained by advancing age, medical problems, or mobility issues such as degeneration of the spine or other joints.

Our radiological evaluation revealed osseointegration of all femoral stems in our study. The radiolucent lines suggestive of stress shielding were non-progressive throughout the follow-up with no pain or limitation of function. Calcar atrophy caused by stress shielding in uncemented THA has shown slow progressive recovery over the years, without significant clinical relevance [19]. The low degree of proximal bone loss in our study indicates no significant net transfer of stress from proximal to distal, but rather a physiological weight distribution from the stem to the femoral bone after osseointegration.

There were eight (2%) intra-operative femoral fractures, all secured by a cerclage wire. The prevalence of intra-operative femoral fractures increases in advanced age groups, females, and conditions such as osteoporosis [20]. The risk is higher in uncemented stems and can be related to stem design, with one study showing a relation between the size of this HAC stem and the occurrence of intra-operative fractures. Furthermore, a systematic review showed a higher risk of fractures with uncemented prostheses, especially single and double-wedged tapered stems [21, 22]. There was no statistical significance in our study between the occurrence of intra-operative fractures and age or sex.

Our study had two (0.5%) dislocations, with one requiring revision for component malalignment. The fourth-generation ceramic head 36 mm was used in all cases, which may explain the low dislocation rate, due to the biomechanical advantages of the large head with increased range of motion and jumping distance [23]. The dislocation rates were lower than in other studies evaluating the same stem; this can be attributed to the use of different bearings and smaller head sizes in those studies [4, 13, 15]. The head diameter may not achieve stability alone; implant alignment and soft tissue tension are equally or even more important than head diameter [24].

Our patients were asked specifically about thigh pain; two patients (0.5%) reported thigh pain, describing it as intermittent, mild, and not interfering with their activities of daily living. Their radiographs showed no evidence of loosening or stress shielding. The low prevalence of thigh pain can be explained by the rapid osseointegration of the stem, the stem design that relies on metaphyseal fitting without the need for stem-fitting in the diaphysis, and the lower modulus of elasticity of the titanium implant [25].

We acknowledge some limitations in our study. First, the few pre-operative MDP scores for patient activity. However, there was an accurate record of scores from the time of surgery with a mean pre-operative and post-operative OHS. The VAS and PROM scores were consistently used as patient-reported tools to augment our clinical data. Second, there was a lack of reports on wear measurements. In long-term studies, wear measurements are vital to understanding the prognosis of the prosthesis.

In conclusion, our study had 100% survival with full osseointegration at the final follow-up, no stem failures due to aseptic loosening, and excellent PROMs at a mean follow-up of 9.39 years (range, 4–14.5 years). We assessed the hips with a unified bearing surface with the same femoral head size and the same acetabular component to avoid any variability in wear patterns and rates. Radiological evaluations showed complete osseointegration with minimal stress shielding.

## Data Availability

All research data is available upon request.

## References

[1] Scott CH, MacDonald DJ, Howie CR (2019) “Worse than death” and waiting for a joint arthroplasty. Bone Joint J 101, 941–950. 31362549 10.1302/0301-620X.101B8.BJJ-2019-0116.R1 PMC6681678

[2] Johnsen SP, Sørensen HT, Lucht U, Søballe K, Overgaard S, Pedersen AB (2006) Patient-related predictors of implant failure after primary total hip replacement in the initial, short- and long-terms. A nationwide Danish follow-up study including 36,984 patients. J Bone Joint Surg Br 88, 1303–1308. 17012418 10.1302/0301-620X.88B10.17399

[3] Chandler HP, Reineck FT, Wixson RL, McCarthy JC (1981) Total hip replacement in patients younger than thirty years old: a five-year follow-up study. J Bone Joint Surg Am 63-A, 1426–1434. 7320033

[4] Syed MA, Hutt NJ, Shah N, Edge AJ (2015) Hydroxyapatite ceramic-coated femoral components in young patients followed up for 17 to 25 years: an update of a previous report. Bone Joint J 97-B, 749–754. 26033053 10.1302/0301-620X.97B6.35278

[5] Rahbek O, Overgaard S, Søballe K (2004) Calcium phosphate coatings for implant fixation. In: Fifteen years of clinical experience with hydroxyapatite coatings in joint arthroplasty,Springer, Paris, pp. 35–51. 10.1007/978-2-8178-0851-2_4.

[6] Overgaard S, Bromose U, Lind M, Bünger C, Søballe K (1999) The influence of crystallinity of the hydroxyapatite coating on the fixation of implants. J Bone Joint Surg Br 81-B, 725–731. 10.1302/0301-620x.81b4.9282 10463753

[7] Karia M, Logishetty K, Johal H, Edwards TC, Cobb JP (2023) 5 year follow up of a hydroxyapatite coated short stem femoral component for hip arthroplasty: a prospective multicentre study. Sci Rep 13, 17166. 37821511 10.1038/s41598-023-44191-7 PMC10567683

[8] Ahmad MA, Xypnitos FN, Giannoudis PV (2011) Measuring hip outcomes: common scales and checklists. Injury 42, 259–264. 21163481 10.1016/j.injury.2010.11.052

[9] EuroQol Group (1990) EuroQol – a new facility for the measurement of health-related quality of life. Health Policy 16, 199–208. 10109801 10.1016/0168-8510(90)90421-9

[10] Muir SW, Al-Ahaideb A, Huckell J, Johnson MA, Johnston DBC, Beaupre LA (2011) Radiographic assessment of uncemented total hip arthroplasty: reliability of the Engh Grading Scale. Can J Surg 54, 185–188. 21609518 10.1503/cjs.002610 PMC3104312

[11] Hug KT, Alton TB, Gee AO (2015) Classifications in brief: Brooker classification of heterotopic ossification after total hip arthroplasty. Clin Orthop Relat Res 473, 2154–2157. 25427427 10.1007/s11999-014-4076-x PMC4419015

[12] Sandiford N, Doctor C, Rajaratnam SS, Ahmed S, East DJ, Miles K, et al. (2013) Primary total hip replacement with a Furlong fully hydroxyapatite-coated titanium alloy femoral component: results at a minimum follow-up of 20 years. Bone Joint J 95-B, 467–471. 23539697 10.1302/0301-620X.95B4.30445

[13] Baltopoulos P, Tsintzos C, Papadakou E, Karagounis P, Tsironi M (2008) Hydroxyapatite-coated total hip arthroplasty: the impact on thigh pain and arthroplasty survival. Acta Orthop Belg 74, 323–331. 18686456

[14] Hamilton WG, McAuley JP, Blumenfeld TJ, Lesko JP, Himden SE, Dennis DA (2015) Midterm results of delta ceramic-on-ceramic total hip arthroplasty. J Arthroplasty 30, 110–115. 26122108 10.1016/j.arth.2015.02.047

[15] Piolanti N, Neri E, Bonicoli E, Parchi PD, Marchetti S, Manca M, et al. (2021) Use of a plasma-sprayed titanium-hydroxyapatite femoral stem in hip arthroplasty in patients older than 70 years. Is cementless fixation a reliable option in the elderly? J Clin Med 10, 4735. 34682858 10.3390/jcm10204735 PMC8540300

[16] Coulomb R, Mansour J, Essig J, Asencio G, Kouyoumdjian P (2022) Clinical results at 10 years of minimum follow-up with the ABG 2 hip arthroplasty, matched with ceramic-on-ceramic bearings. SICOT J 8, 32. 35969123 10.1051/sicotj/2022032 PMC9377216

[17] Willburger RE, Heukamp M, Lindenlaub P, Efe T, Peterlein CD, Schüttler KF (2020) Excellent midterm survival and functional outcomes of a fully hydroxyapatite-coated cementless stem: first results of a prospective multicenter study. Arthroplast Today 6, 201–205. 32577462 10.1016/j.artd.2020.01.009 PMC7303481

[18] Orthopaedic Data Evaluation Panel (ODEP) (2024) Furlong HAC femoral stem – Waldemar Link. Available at: https://www.odep.org.uk/product/furlong-hac-coated [Accessed 04 January 2024].

[19] Karachalios T, Tsatsaronis C, Efraimis G, Papadelis P, Lyritis G, Diakoumopoulos G (2004) The long-term clinical relevance of calcar atrophy caused by stress shielding in total hip arthroplasty: a 10-year, prospective, randomized study. J Arthroplasty 19, 469–475. 15188106 10.1016/j.arth.2003.12.081

[20] Zhu Y, Chen W, Sun T, Zhang X, Liu S, Zhang Y (2015) Risk factors for periprosthetic fracture after total hip arthroplasty: A systematic review and meta-analysis. Scandinavian J Surgery 104(3), 139–145. 10.1177/1457496914543979 25053584

[21] Barlas KJ, Ajmi QS, Bagga TK, Howell FR, Roberts JA, Eltayeb M (2008) Association of intra-operative metaphyseal fractures with prosthesis size during hemiarthroplasty of the hip. J Orthop Surg (Hong Kong) 16, 30–34. 18453655 10.1177/230949900801600108

[22] Carli A, Negus JJ, Haddad FS (2017) Hip arthroplasty: Avoiding and managing problems periprosthetic femoral fractures and trying to avoid them: What is the contribution of femoral component design to the increased risk of periprosthetic femoral fracture? Bone Joint J 99-B, 50–59. 28042119 10.1302/0301-620X.99B1.BJJ-2016-0220.R1

[23] Jameson SS, Lees D, James P, Serrano-Pedraza I, Partington PF, Muller SD, et al. (2011) Lower rates of dislocation with increased femoral head size after primary total hip replacement: A five-year analysis of NHS patients in England. J Bone Joint Surg Br 93, 876–880. 21705556 10.1302/0301-620X.93B7.26657

[24] Sariali E, Lazennec JY, Khiami F, Catonné Y (2009) Mathematical evaluation of jumping distance in total hip arthroplasty: Influence of abduction angle, femoral head offset, and head diameter. Acta Orthop 80, 277–282. 19421906 10.3109/17453670902988378 PMC2823207

[25] Rajaratnam SS, Jack C, Tavakkolizadeh A, George MD, Fletcher RJ, Hankins M, et al. (2008) Long-term results of a hydroxyapatite-coated femoral component in total hip replacement: a 15- to 21-year follow-up study. J Bone Joint Surg Br 90, 27–30. 18160495 10.1302/0301-620X.90B1.19731

